# Inhibition of PTP1B blocks pancreatic cancer progression by targeting the PKM2/AMPK/mTOC1 pathway

**DOI:** 10.1038/s41419-019-2073-4

**Published:** 2019-11-19

**Authors:** Qi Xu, Ning Wu, Xiangqian Li, Chuanlong Guo, Chao Li, Bo Jiang, Huaizhi Wang, Dayong Shi

**Affiliations:** 1 State Key Laboratory of Microbial Technology, Shandong University, Jinan, 250100 Shandong China; 2 Key Laboratory of Experimental Marine Biology, Institute of Oceanology, Chinese Academy of Sciences, Qingdao, China; 3 Laboratory for Marine Drugs and Bioproducts, Qingdao National Laboratory for Marine Science and Technology, Qingdao, China; 4 The University of Chinese Academy of Sciences, Beijing, China; 5 Institute of Hepatopancreatobiliary Surgery, Southwest Hospital, Third Military Medical University, Chongqing, 400038 China

**Keywords:** Cancer therapy, Oncogenes

## Abstract

Pancreatic cancer is a highly malignant cancer and lacks effective therapeutic targets. Protein-tyrosine phosphatase 1B (PTP1B), a validated therapeutic target for diabetes and obesity, also plays a critical positive or negative role in tumorigenesis. However, the role of PTP1B in pancreatic cancer remains elusive. Here, we initially demonstrated that PTP1B was highly expressed in pancreatic tumors, and was positively correlated with distant metastasis and tumor staging, and indicated poor survival. Then, inhibition of PTP1B either by shRNA or by a specific small-molecule inhibitor significantly suppressed pancreatic cancer cell growth, migration and colony formation with cell cycle arrest in vitro and inhibited pancreatic cancer progression in vivo. Mechanism studies revealed that PTP1B targeted the PKM2/AMPK/mTOC1 signaling pathway to regulate cell growth. PTP1B inhibition directly increased PKM2 Tyr-105 phosphorylation to further result in significant activation of AMPK, which decreased mTOC1 activity and led to inhibition of p70S6K. Meanwhile, the decreased phosphorylation of PRAS40 caused by decreased PKM2 activity also helped to inhibit mTOC1. Collectively, these findings support the notion of PTP1B as an oncogene and a promising therapeutic target for PDAC.

## Introduction

Pancreatic ductal adenocarcinoma (PDAC) is one of the most fatal cancers in the world with the worst prognosis, and its incidence has increased in recent years^1^. To date, the rate of pancreatic cancer incidence nearly equals its mortality due to diagnosis at later stage and the lack of early detection markers^2^. According to an estimate by the American Cancer Society, PDAC will be the second leading cause of cancer-related death in the USA by 2030^3^. Unfortunately, the effect of standard therapies, including surgery, systemic chemotherapy, and radiation therapy, on patient survival is merely modest, leading to a poor 5-year survival rate of only 6%^4^. As a result, there is a critical need to explore potential therapeutic targets and elucidate the biological mechanism underlying pancreatic tumorigenesis and cancer progression, and develop novel therapeutic strategies against PDAC.

Aberrant interplay between tyrosine kinases and protein-tyrosine phosphatases disrupts the degree of signaling within many signal transduction pathways that control cell growth, differentiation and metabolism^5^. Based on these findings, protein-tyrosine phosphatase 1B (PTP1B) encoded by PTPN1 is attracting considerable interest with enormous therapeutic potential^6^. Thus far, PTP1B has been validated as a target for therapeutic intervention in diabetes and obesity^7^, and is associated with dendritic cell-based cancer immunotherapy^8^. Additionally, accumulating evidence has indicated that PTP1B is also involved in the progression of many cancers^9^. Interestingly, the role of PTP1B in tumorigenesis is variable and depends on the tumor type^10^. PTP1B functions as a tumor promoter in gastric cancer, prostate cancer, colorectal cancer, non-small cell lung cancer and hepatocellular carcinoma and correlates with poor prognosis by deregulating oncogenes or tumor suppressors^9^. In breast cancer, the role of PTP1B in oncogenesis has been clearly clarified, and treatment with a PTP1B inhibitor significantly delayed breast tumor development^11^. It is because of the significant role of PTP1B in cancer that a PTP1B inhibitor—MSI-1436C, a potential drug for diabetes, was previously tested in a phase I clinical trial for the treatment of metastatic breast cancer^12^. Conversely, PTP1B is downregulated and acts as a negative regulator of BRK and IGF-1R signaling in ovarian cancer cells^13^, and acts as a tumor suppressor in B-cell lymphoma^14^. Until now, no studies have reported the role of PTP1B in PDAC proliferation and progression, although both a tumor suppressor and an oncogene function of PTPN1 have been proposed. Because PTP1B is the target of diabetes mellitus and obesity, which are two established risk factors for PDAC^3^, it is important to determine the roles of PTP1B in pancreatic cancer.

Hence, in this study, we discovered that PTP1B is overexpressed in pancreatic cancer tissues and is required for maintaining pancreatic cancer cell proliferation and tumor growth in vitro and in vivo. In addition, we identified that the PKM2/AMPK/mTOC1 signaling pathway is essential for PTP1B to regulate cell growth.

## Materials and methods

### Human tissue specimens

Two groups of human tissues (group 1: 103 pancreatic cancer samples and 13 normal pancreas samples, and group 2: fifteen pancreatic cancer samples and corresponding paracancer tissue) were obtained from Southwest Hospital, Third Military Medical University between October 2010 and June 2016. Patient follow-up visits were continued until June 20 2016, and the overall survival (OS) of the PDAC patients was measured from date of PDAC surgery until date of death or last follow-up. All human sample collection procedures were approved by the relevant Ethics Committee of the Southwest Hospital, Third Military Medical University. Informed consent was obtained for all cases before surgery.

### Cell culture

PANC-1, MIA-PaCa-2, SW-1990, AsPC-1, PANC-28, BxPC-3 cell lines were obtained from the Chinese Academy of Science Cell Bank (Shanghai, China). HPDE cell line was purchased from BeNa Culture Collection (Beijing, China). All cell lines in the experiments were validated by STR DNA analysis and were negative for mycoplasma. HPDE was cultured in RPMI 1640 (Hyclone). SW-1990 was maintained in L-15 Medium (Hyclone). PANC-1, MIA-PaCa-2, AsPC-1, PANC-28, BxPC3 were grown in DMEM (Hyclone). All media were supplemented with 10% fetal bovine serum (Gibco) and 1% penicillin/streptomycin. All cells were maintained at 37 °C in a humidified atmosphere with 5% CO_2_ except SW1990 which cultured at 37 °C in a CO_2_-free incubator.

### Materials and antibodies

LXQ46 (3,4-dibromo-5-(5-(4-(4-ethoxyphenoxy)phenyl)oxazol-2-yl)benzene-1,2-diol) was synthesized and identified by our lab (purity 98%). Primary antibodies used in this study: PTP1B (#133259) were obtained from Santa Cruz Biotechnology (Santa Cruz, CA, USA). PARP (#9532), Bcl-xL (#2764), Phospho-AMPK (Thr172, #2535), AMPK (#2793S), Phospho-Akt (Ser473, #4060), Akt (#4685), Phospho-ERK (Thr202/Tyr204, #4370S), ERK (#4696S), Phospho-PRAS40 (#2640) and Ki67 (#9027) were purchased from Cell Signaling Technology (Danvers, MA, USA). Bax (#32503), Bcl-2 (#32124), CDK2 (#32147), CDK4 (#108357), CDK6 (#124821), Cyclin D1(#134175), Phospho-Src (Tyr529, #32078), Src (#47405), Phospho-PI3K (#182651), PI3K (#86714), PRAS40 (#151719) were purchased from Abcam (Cambridge, MA, USA). Phospho-p70 S6 Kinase (Thr389/412, #3228) and p70 S6 Kinase (#6226), Phospho-PKM2 (Tyr105 #7771), PKM2 (#5234) were purchased from Affinity Biosciences (Cincinnati, OH, USA). β-actin antibody and all secondary antibodies were obtained from Proteintech Group (Wuhan, China).

### Lentiviral preparation and infection

#### PTPN1 short hairpin RNA (shRNA)

The recombinant vector (pGLV3-GFP& puro-PTPN1) contained different shRNA sequences targeting PTPN1 was termed LV3-shPTP1B (GenePharma, Shanghai, China). The negative control vector (pGLV3-GFP& puro-Con) contained a nonsense shRNA was termed LV3-shCon (GenePharma, Shanghai, China). The sequences of the shRNAs are as follows: shPTP1B-1, 5′- GGAAGAGACCCAGGAGGATAA-3′; shPTP1B-2, 5′-GGAAATGCAGGGAGTTCTTCC-3’; shCon, 5′- TTCTCCGAACGTGTCACGT-3’. For the generation of lentivirus, 293T producer cells were transfected with optimized packaging plasmid (pGag/Pol, pRev, pVSV-G) along with LV3-shPTP1B or LV3-shCon vector by lipofectamine (Invitrogen). After 72 h, virus-containing medium was collected and used for infection of target cells in the presence of polybrene. Then, the stable cells were selected by puromycin (5 μg/mL).

#### Preparation of lentivector-PTP1B overexpression construct

The full-length coding sequence of PTP1B was amplified. The PCR product was digested with NotI and NsiI and ligated to pGLV-EF1a-GFP (LV-4, GenePharma, Shanghai, China). The recombinant vector pGLV-GFP-PTP1B was named LV4-PTP1B. Then, the generation and propagation lentivirus were carried out as described above.

### MTT assays

#### Cell growth assay

After transfected, 2 × 10^3^ cells were seeded in per well in a 96-well plate and the total cell numbers were determined by MTT assay. MTT (Solarbio, Beijing, China) was added to each well every 48 h and incubated with cells for 4 h. Then the supernatants were discarded and MTT formazan crystals were dissolved by adding 150 μL DMSO per well. The optical density (OD) of each well was measured with a microplate reader at 490 nm.

#### Cell cytotoxicity assay

Depending on cell line, 5000–8000 cells were plated in a 96-well plate and incubated at 37 °C overnight before drug exposure. Then cells were treated with the LXQ46 to achieve final concentrations (0, 2.5, 5, 10, 20 μM) for 48 h. The cells viability was determined by MTT assay, as mentioned above.

### Colony formation assay

After digestion and count, PANC-1 or MIA-PaCa-2 cells which were infected with lentiviral or treated with LXQ46 with final concentrations (0, 5, 10, 15 μM) for 48 h were seeded in 6-well plates at 1000 cells/well and maintained in media containing 10% FBS. Next, plates were incubated under standard conditions for 10–14 days. Colonies in each well were fixed with 4% paraformaldehyde (PFA), stained with 0.5% crystal violet, counted, and photographed.

### Transwell migration assay

The cells were digested and then washed twice and resuspended with serum-free DMEM. The cell density was adjusted to 5 × 10^5^ cells/ml and 200 μl diluted cells were seeded into the upper chambers of Costar 24-transwell assay plates, and 600 μl of 10% FBS DMEM was added to the lower chamber as a chemoattractant. After 24 h of incubation, the filter was washed with PBS, swabbed using cotton buds, and then fixed with 4% paraformaldehyde for 30 min. Then, the upper chamber was stained by 0.5% crystal violet for 10 min and rinsed with distilled water. After this, the chambers were photographed using microscope. The number of cells in each image was counted by the Image J software.

### Flow cytometry analysis

Cell cycle distribution was analyzed by flow cytometry with DNA staining. Cells (4 × 10^5^ cells/well) were synchronized by culturing them in serum-free media for 24 h followed by the indicated treatments. Subsequently, cells were harvested, washed twice with PBS, and fixed in cold 70% ethanol at −20 °C overnight. Then the cells were washed and incubated with cold PBS containing 20 µg/mL RnaseA (Sigma, St. Louis, MO, USA) and 50 µg/mL propidium iodide (PI, Sigma, St. Louis, MO, USA) for 30 min at 37 °C before being analyzed on flow cytometer (Becton Dickinson, Franklin Lakes, NJ, USA). The percentage of cells in the various stages of the cell cycle was determined using the ModFit LT software (Verity Software House, Topsham, ME, USA).

### Western blotting analysis

Cultured cells and tumor tissues from nude mice were lysed with ice-cold RIPA buffer containing freshly added PMSF lysis buffer. The total protein was quantified using the BCA Protein Assay Kit (Beyotime, Guangzhou, China). Cell lysates were separated by SDS-PAGE and transferred onto a PVDF membrane (Millipore, Billerica, MA, USA). Membranes were blocked in 5% non-fat milk in 1× Tris-buffered saline containing 0.05% Tween 20 (TBST) for 1 h at room temperature, and then incubated with primary antibodies overnight at 4 °C, followed by incubation with HRP-conjugated secondary antibodies for 1 h at room temperature. The bands were visualized using enhanced chemiluminescence (ECL) reagent (Bio-Rad, Hercules, CA) with the ChemiDoc XRS imaging system (Bio-Rad, USA) and the intensities were analyzed by the Image J public domain software from the National Institutes of Health (Bethesda, MD, USA).

### Xenograft assay

All experimental procedures involving animals were conducted in accordance with all appropriate regulatory standards under protocol HAIFAJIZI-2013-3 (approval date: 2013-12-09) approved by the Animal Care and Use Committee of Institute of Oceanology, Chinese Academy of Sciences.

#### Xenograft model of PTP1B knockdown

5 × 10^6^ viable PANC-1 cells infected with LV3-shPTP1B-1 or LV3-shCon were resuspended in 100 μl PBS and subcutaneously inoculated into the front-right armpit of 5–6 weeks old BALB/c nude mice (Vital River Laboratory Animal Technology Co., Ltd, Beijing, China) with six mice (three females and three males) in each group. Tumor volumes (length × width^2^/2) and body weight were monitored every two days in each group. All the mice were sacrificed 24 days after inoculation, tumors were excised, weighed and photographed.

#### Xenograft model of PTPT1B inhibition

A total of 4 × 10^6^ PANC-1 cells/mice were subcutaneously inoculated into the right flank of nude mice (5–6 weeks old) to initiate tumor growth. One week later, when the tumor size reached approximately 100 mm^3^, mice were randomized into three groups (*n* = 6). Negative control group was treated with vehicle and other groups were treated with LXQ46 (50 mg/kg and 100 mg/kg) intraperitoneally once daily for 18 days. The tumor volumes (length × width^2^/2) and mice body size were measured once every three days. At the end of the experiment, mice were euthanized and tumors were isolated, weighed and photographed.

### Immunohistochemistry

Human tissues and tumor xenograft tissues were fixed with formalin and embedded in paraffin. Then 3–4 μm thick sections of the tissues were prepared for analysis. For immunohistochemistry, the paraffin sections were de-paraffinized and rehydrated, and were incubated with primary antibodies at 4 °C overnight. After three washes with PBS, the sections were incubated with secondary antibodies at room temperature for 30 min and treated with streptavidin-HRP. For the histological analysis, specimens were stained with haematoxylin and eosin (H&E) to identify morphological changes. Tissue sections were observed using a light microscope (Nikon, Tokyo, Japan). The staining score of protein expression was quantified as reported previously^15^.

### Statistical analysis

Each experiment was repeated three times. Unless otherwise stated, all data are presented as mean ± SEM values. Comparison of two groups was performed by two-tailed Student's *t* test (SPSS 19.0, USA). Comparison among three or more groups were analyzed with one-way ANOVA followed by Tukey’s *post hoc test*. The Kaplan–Meier method was used to calculate the patient survival probability. The Chi-square test was used to evaluate the relationship between PTP1B and characteristics of patients with PDAC. The GraphPad Prism 6.0 software was used for statistical evaluations. Statistically significant difference was defined as *P* < 0.05.

## Result

### PTP1B expression is highly elevated in PDAC and correlates with poor survival

**Fig. 1:**
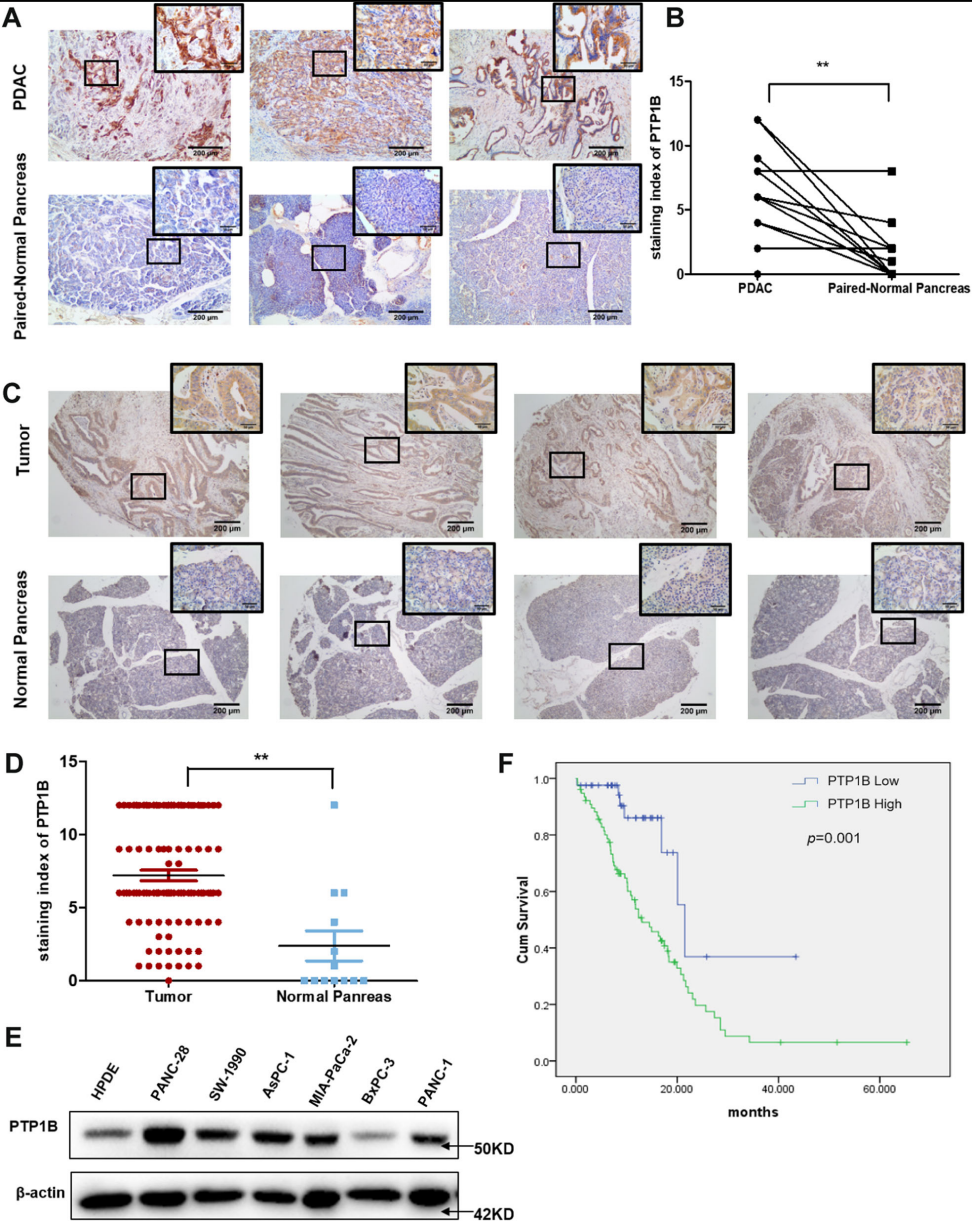
PTP1B overexpression indicated the poor prognosis for pancreatic cancer patients. **a**, **c** PTP1B expression in tissues were detected by immunohistochemistry (IHC) assays (scale bar, 200 μm and 50 μm). **b**, **d** Staining index of PTP1B in tumor and nonneoplastic tumor tissue. **e** PTP1B protein levels in normal pancreatic ductal cell line (HPDE) and different pancreatic cell lines were examined by Western blotting. **f** Kaplan–Meier analysis of the correlation between PTP1B level and overall survival of pancreatic cancer patients (log-rank test *p* = 0.001).

To understand the clinical relevance of PTP1B in PDAC, we examined PTP1B expression in paired PDAC and adjacent normal pancreatic tissues from 15 patients. As shown in Fig. 1a, b, PTP1B expression was highly elevated in 13 tumor parts compared with the nonneoplastic surrounding counterparts. By using tissue microarray (13 samples of normal pancreas and 103 samples of PDAC tissues), we also observed that PTP1B was overexpressed in the tumor samples but rarely expressed in normal tissues (Fig. 1c, d). In conclusion, the expression of PTP1B was significantly higher in tumor tissues than in nontumor tissues. We then used immunoblotting to compare PTP1B expression in a normal pancreatic ductal cell line (HPDE) and a series of PDAC cell lines. The results demonstrated that PTP1B expression was markedly increased in PDAC cell lines (Fig. 1e). Furthermore, we investigated the correlation of PTP1B expression level with the prognosis of patients with PDAC. The results showed that the overall survival time of patients with low PTP1B levels was significantly longer than that of patients with high PTP1B levels (Fig. 1f). In addition, the relationships between PTP1B and clinicopathological parameters were analyzed and presented in Table 1 and Supplementary Table 1. We discovered that PTP1B expression is positively correlated with pancreatic cancer distant metastasis (*p* = 0.002) and tumor staging (*p* = 0.004). Taken together, these data confirmed our hypothesis that PTP1B is highly associated with PDAC progression and may be considered a potential biomarker for PDAC diagnosis.

**Table 1:** Associations between PTP1B and clinical characteristics of PDAC.

Characteristic	*n*	PTP1B		χ2	*p*
		High	Low		
*Gender*					
Male	71	47	24	0.07	0.791
Female	47	30	17		
Age					
<60	49	29	20	1.362	0.243
≥60	69	48	21		
*Tumor (Topography)*					
T1,<2 cm	23	11	12	4.508	0.212
T2, ≥2 cm, ≤4	67	48	19		
T3, >4	21	13	8		
T4, invasion	7	5	2		
*Lymph node metastasis (N)*					
NO	80	50	30	0.831	0.362
YES	38	27	11		
*Distant metastasis (M)*					
NO	107	66	41	9.985	0.002
YES	11	11	0		
*Differentiation*					
Low	28	18	10	0.18	0.914
Medium	86	56	30		
High	4	3	1		
*Tumor staging*					
I	42	20	22	13.535	0.004
II	59	32	27		
III	7	5	2		
IV	10	10	0		

### PTP1B deficiency inhibits PDAC cell proliferation, cell cycle progression and migration

**Fig. 2:**
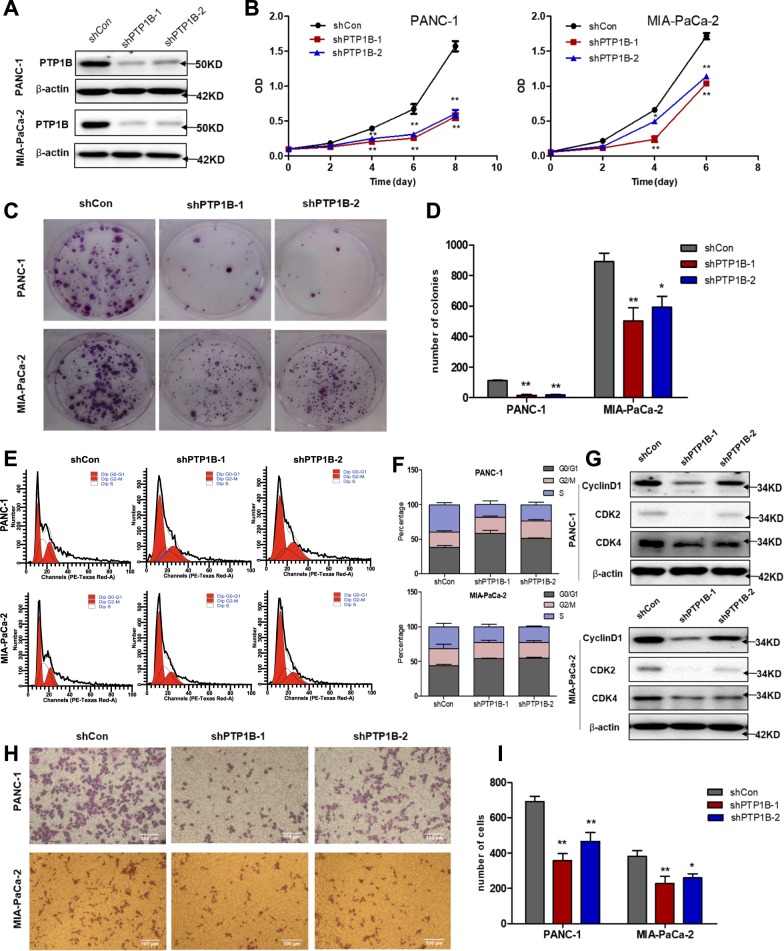
PTP1B is required for PDAC cell growth. **a** Significant knockdown of PTP1B protein by shRNAs in PANC-1 and MIA-PaCa-2 cells was detected by Western blotting. LV3-shPTP1B1 and LV3-PTP1B2, which had different PTP1B targeting sequences, were used in this study. **b** PTP1B knockdown inhibited pancreatic cancer cells growth. Cell growth was measured by MTT assay. Each time point has four repeats. **c**, **d** Colony formation assays showed PTP1B silencing decreased cell proliferation. The representative images were shown in (**c**). The quantitative analysis was shown in (**d**). **e**, **f** PTP1B knockdown induced cell cycle arrest in G0/G1 phase, which was analyzed by PI staining using flow cytometry. **g** A set of signaling molecules related with G1-S transition were detected by Western blotting, and found to be downregulated in PTP1B knockdown PANC-1 and MIA-PaCa-2 cells. **h**, **i** Transwell migration assay indicated that the knockdown of PTP1B significantly reduced the metastasis of PDAC cells. The representative images were shown in (**h**) (scale bar, 100 μm). Quantitative analysis was shown in (**i**). All the quantitative data are represented as mean ± SEM of three independent experiments and *p *<* 0.05, **p *<* 0.01 versus control group.

Next, to assess whether PTP1B is required for maintaining pancreatic cancer cell growth, we used specific shRNAs to knockdown PTP1B in PANC-1 and MIA-PaCa-2 cells. Compared with scrambled shRNA, both shPTP1B-1 and shPTP1B-2 significantly reduced PTP1B expression in stable cell lines (Fig. 2a). Then, 72 h after LV3-shRNAs transfection, the cell number was significantly lower in shPTP1B-treated cells than in the control ones (Supplementary Fig. 1). Moreover, MTT assay results showed that silencing PTP1B led to significant inhibition of PDAC cell proliferation (Fig. 2b). As demonstrated by colony formation, PTP1B knockdown also suppressed cancer cell growth (Fig. 2c, d). In addition, flow cytometry analysis showed that silencing PTP1B dramatically increased the G0/G1 ratio and reduced the percentage of cells in S phase (Fig. 2e, f), indicating that the loss of PTP1B induced cell cycle arrest in G0/G1 phase. Accordingly, several cell cycle regulators of the G1-S transition, CDK2, CDK4 and Cyclin D1, were downregulated in PTP1B knockdown cells compared with the levels in the control cells (Fig. 2g). Notably, the reduced growth upon silencing PTP1B was mainly due to decreased cell proliferation, not apoptosis, because we did not find substantial increase of cleaved PARP and Bax or decrease of Blc-2 and Bcl-xL in PTP1B deficient cells (Supplementary Fig. 2a). Additionally, given the positive relationship between PTP1B and distant metastasis of PDAC mentioned above (Table 1), we explored the role of PTP1B in PDAC cell movement. Thus, transwell assay was performed, which revealed that knocking down PTP1B inhibited the migratory ability of cancer cells (Fig. 2h, i). All these effects caused by silencing PTP1B were positively correlated with the efficiency of PTP1B knockdown, indicating that PTP1B contributes to the oncogenic phenotypes of pancreatic cancer cells.

### PTP1B overexpression promotes proliferation and migration of pancreatic cancer cell

**Fig. 3:**
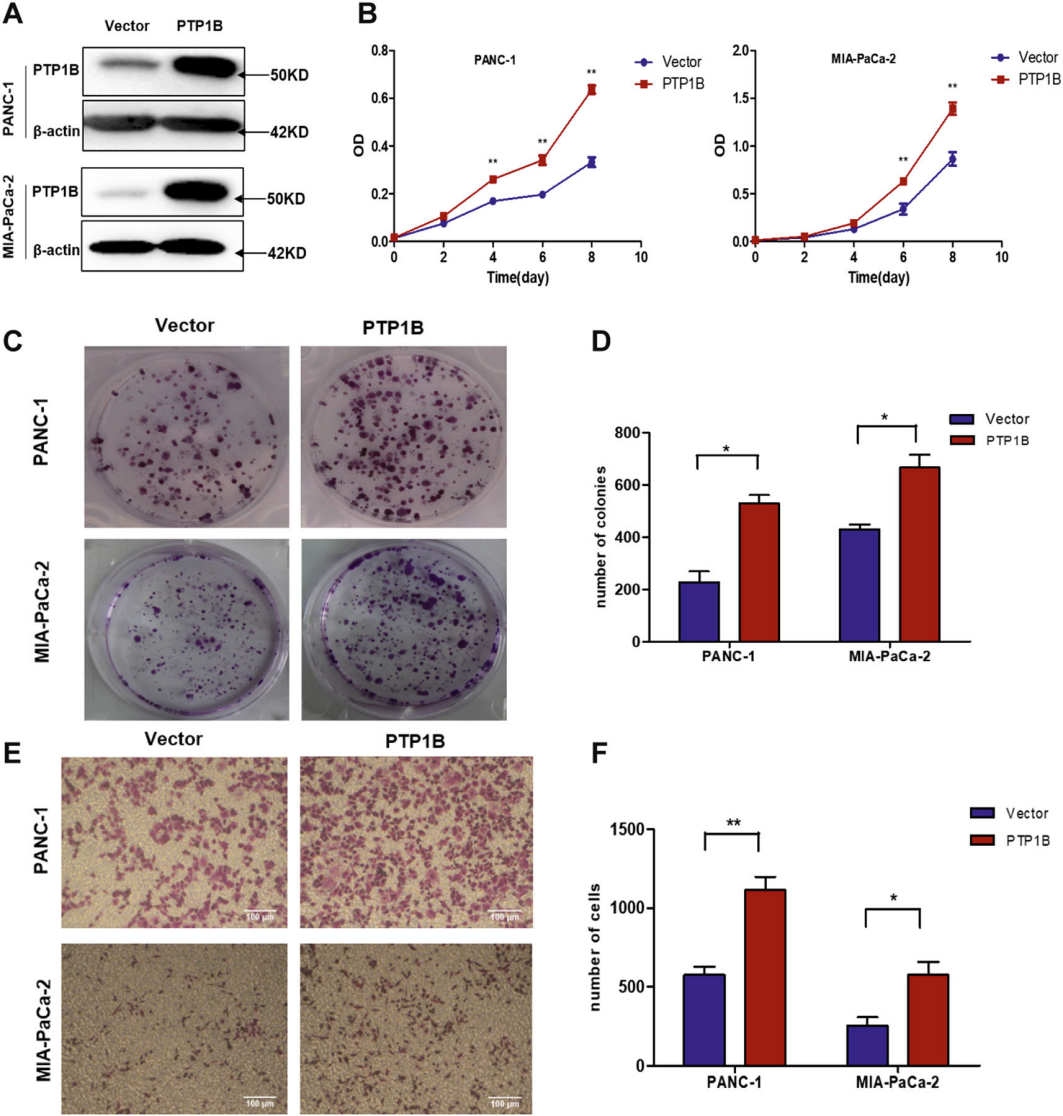
Upregulation of PTP1B promotes proliferation and migration of pancreatic cancer cells. **a** Western blotting showed PTP1B was upregulated in PANC-1 and MIA-PaCa-2 cells after transfected with LV4-PTP1B. **b**–**d** After transfection with LV4-PTP1B, the proliferation and growth of PDAC cell lines were detected by MTT assay and colony formation assay. The cell growth curves of PANC-1 and MIA-PaCa-2 were shown in (**b**). The representative images and quantitative analysis of colony formation assay were shown in (**c**, **d**). **e**, **f** The migration ability of pancreatic cancer cell was shown by transwell assay. Representative images were shown in (**e**) (scale bar, 100 μm). Quantitative analysis was shown in (**f**). All experiments were performed in triplicate. *p < 0.05, **p < 0.01 versus control group.

Since our findings proved that PTP1B promotes pancreatic cancer progression, we further explored the function of PTP1B overexpression in PDAC cell lines to support our hypothesis. To do this, we transfected PANC-1 and MIA-PaCa-2 cells with LV4-PTP1B to achieve PTP1B overexpression (Fig. 3a). Cell growth and colony formation assays revealed that PTP1B overexpression markedly promoted pancreatic cancer cell proliferation (Fig. 3b–d). A migration assay was also employed to further evaluate the impact of PTP1B overexpression on PDAC cell lines and showed that cells migration was significantly increased by PTP1B overexpression (Fig. 3e, f).

### PTP1B inhibitor leads to suppression of PDAC cell growth

**Fig. 4:**
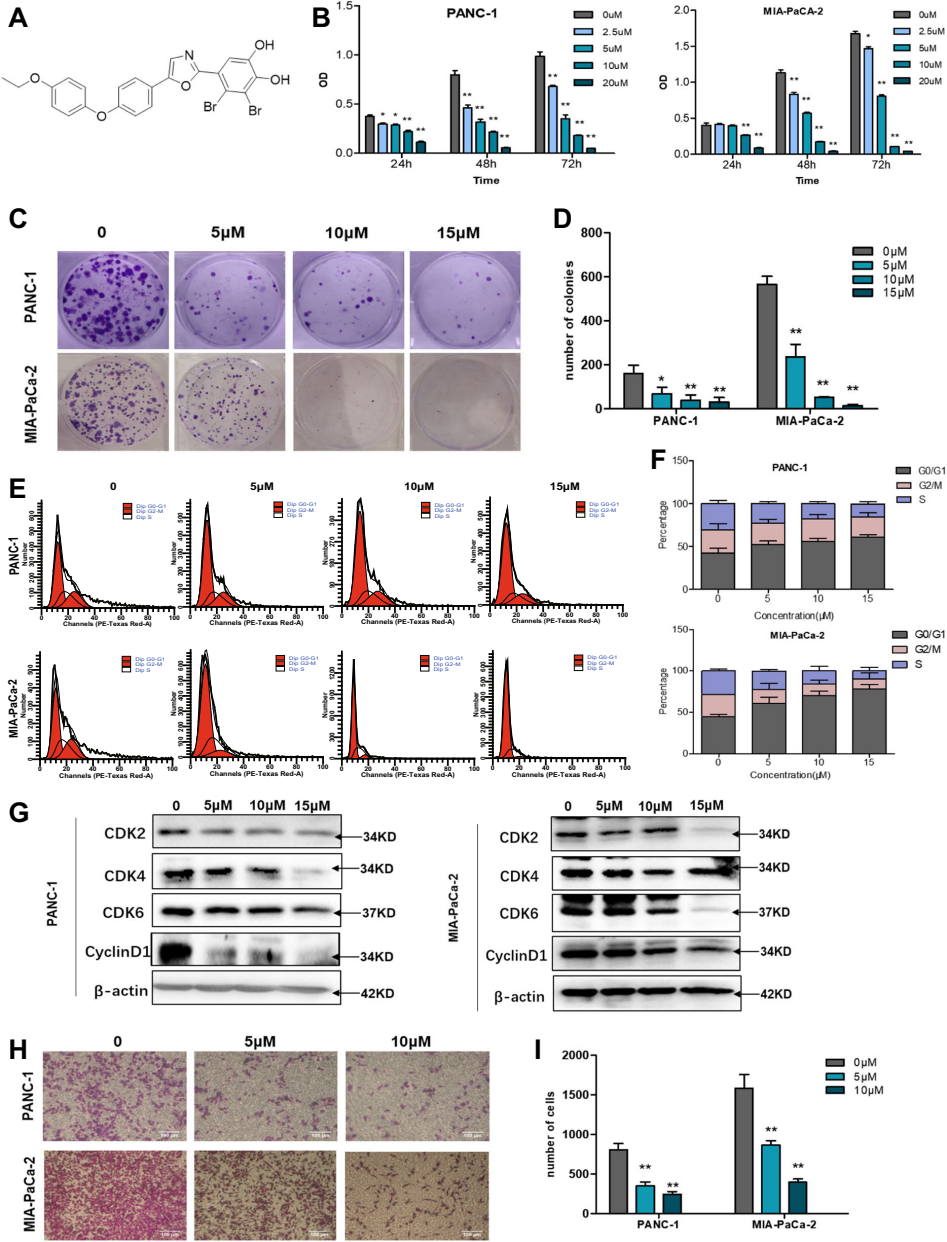
Inhibition of PTP1B activity by small-molecule inhibitor blocks PDAC cell proliferation and migration. **a** Chemical structure of LXQ46. **b** Cell cytotoxicity assay showed LXQ46 inhibited PANC-1 and MIA-PaCa-2 cells proliferation with IC_50_ of 4.169 μM and 4.614 μM, respectively. **c**, **d** Growth of PANC-1 and MIA-PaCa-2 cells treated by indicated concentrations of LXQ46 (0, 5, 10, 15 μM) for 48 h was measured by colony formation assay. Representative images were shown in (**c**). Quantitative analysis was shown in (**d**). **e**, **f** Cell cycle analysis of pancreatic cancer cells following LXQ46 treatment. (**g**) Western blotting analysis of CDK2, CDK4, CDK6, Cyclin D1 in PANC-1 and MIA-PaCa-2 cells after treatment with LXQ46 for 48 h. **h**, **i** The effect of LXQ46 on migration of PDAC cell lines as evaluated by transwell migration assay. Representative images were shown in (**h**) (scale bar, 100 μm). Quantitative analysis was shown in (**i**). Quantification of these data from three independent experiments is shown as mean ± SEM. *p < 0.05 and **p < 0.01 compared with control.

Given the dependency on PTP1B for pancreatic cancer cells proliferation, we used pharmacological approaches to further investigate the pro-survival function of PTP1B in PDAC cells. Therefore, we developed a potent and selective PTP1B inhibitor, LXQ46 (Fig. 4a), and tested its efficacy in PDAC cell lines. LXQ46 is a specific inhibitor of PTP1B with an IC_50_ of 0.190 μM^16^. First, we used Western blotting to determine that LXQ46 had no inhibitory effect on PTP1B expression (Supplementary Fig. 3a). Then, we analyzed the effect of LXQ46 on the proliferation of pancreatic cancer cell lines, including PANC-1, MIA-PaCa-2, SW-1990, AsPC-1, PANC-28, and BxPC3. We observed that LXQ46 significantly reduced the viability of PDAC cell lines in a dose and time-dependent manner (Fig. 4b; Supplementary Fig. 3b–e). Subsequently, we used a clonogenic assay to assess the long-term effect of LXQ46. The results showed that LXQ46 suppressed colony formation in a dose-dependent manner (Fig. 4c, d). Similarly, the growth inhibition of PANC-1 and MIA-PaCa-2 cells was attributed to the obvious G0/G1 phase cell cycle arrest induced by LXQ46 treatment (Fig. 4e, f) rather than apoptosis (Supplementary Fig. 2b). Western blotting analysis further confirmed that LXQ46 induced cell cycle arrest by decreasing the expression of CDK2, CDK4, CDK6, and Cyclin D1 (Fig. 4g). Furthermore, LXQ46 treatment resulted in significant inhibition in PDAC cells migration compared with that in untreated cells (Fig. 4h, i), which was consistent with the effect of PTP1B knockdown on cell migration. Altogether, we identified PTP1B, a positive regulator of pancreatic cancer cell proliferation and migration, as a promising therapeutic target for PDAC.

### Inhibition of PTP1B blocks tumor growth in vivo

**Fig. 5:**
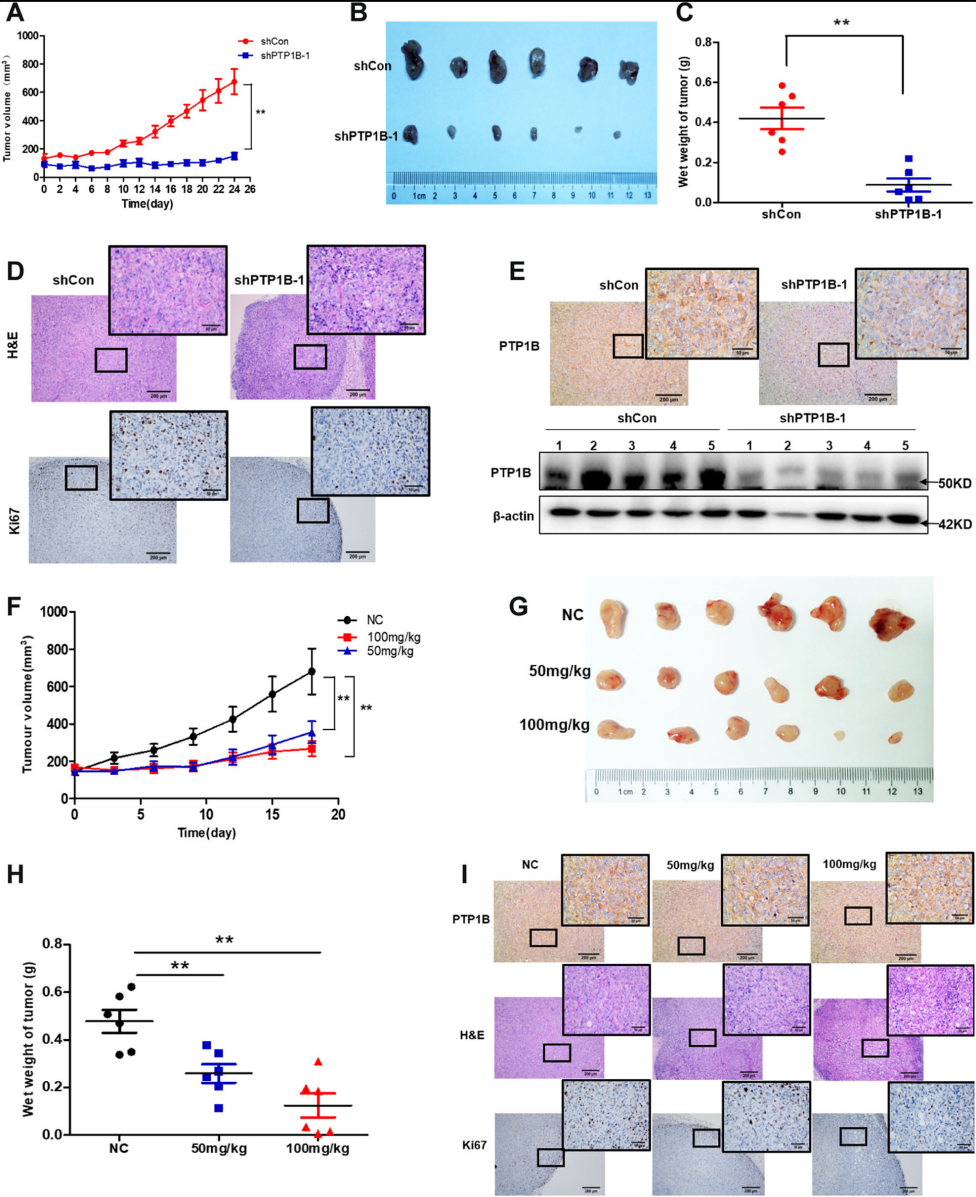
Inhibition of PTP1B suppresses tumor growth. **a**–**e** Loss of PTP1B inhibited PANC-1 cell tumorigenesis in vivo. **a** The changes of tumor volume. **b**, **c** The comparison of tumor size and weight between PTP1B loss group and control group. **d** H&E staining and Ki67 expression in tumor (scale bar, 200 μm and 50 μm). **e** The expression of PTP1B in tumor tissue detected by Western blotting and immunohistochemistry assays (scale bar, 200 μm and 50 μm). **f–i** LXQ46 showed good therapeutic efficacy on pancreatic cancer in mouse xenograft model. BALB/c nude mouse were divided into three groups and treated with vehicle alone or along with LXQ46 (50 mg/kg or 100 mg/kg) intraperitoneally. **f** Tumor volume was measured once every three days for 18 days before mouse were killed. **g**, **h** Image of tumor size and quantitative analysis of the tumor weight. (**i**) H&E staining of tumor and immunohistochemical staining of PTP1B and Ki67 in tumor (scale bar, 200 μm and 50 μm). *p < 0.05, **p < 0.01 versus control group.

Based on the above in vitro findings, we established a xenograft mouse model to further examine the effect of PTP1B on tumor growth in vivo. To this aim, we subcutaneously inoculated PANC-1 cells infected with LV3-shPTP1B-1 or LV3-shCon into nude mice to form tumors. One week after tumor formation, we monitored the growth of transplanted tumors with veneer caliper and observed that tumors growth of PTP1B knockdown was nearly stagnant and much slower than that of the control tumors, indicating PTP1B loss dramatically inhibited tumor growth (Fig. 5a). In agreement with the tumor volumes, the average weight of tumors derived from shPTP1B-1 cells was significantly lower than that of the corresponding control tumors (Fig. 5b, c). Moreover, as shown in Fig. 5d, tumors treated with LV3-shPTP1B-1 displayed an obvious decrease in Ki67 expression, which is indicative of reduced proliferation of tumors in the shPTP1B-1 group. Then, the expression of PTP1B in these tumor tissues were detected by Western blotting and IHC to determine that it remained underexpressed after tumorigenesis (Fig. 5e).

Likewise, pharmacologic inhibition of PTP1B also led to tumor growth suppression in vivo, as evidenced by Fig. 5f–i. Tumors in the control group grew rapidly, whereas LXQ46 treatment (50 mg/kg and 100 mg/kg) significantly blocked tumor growth (Fig. 5f, g). The tumor weight in the LXQ46 treated group was also lower than that of the control group (Fig. 5h). Furthermore, we revealed that the inhibition of cell proliferation in tumor tissues by LXQ46 was not achieved by inhibiting PTP1B expression (Fig. 5i). In summary, all these data suggested that PTP1B is required for tumor growth in vivo, which was concomitant with the in vitro observations.

### PTP1B regulates pancreatic cancer cell growth via the PKM2/AMPK/mTOC1 pathway

**Fig. 6:**
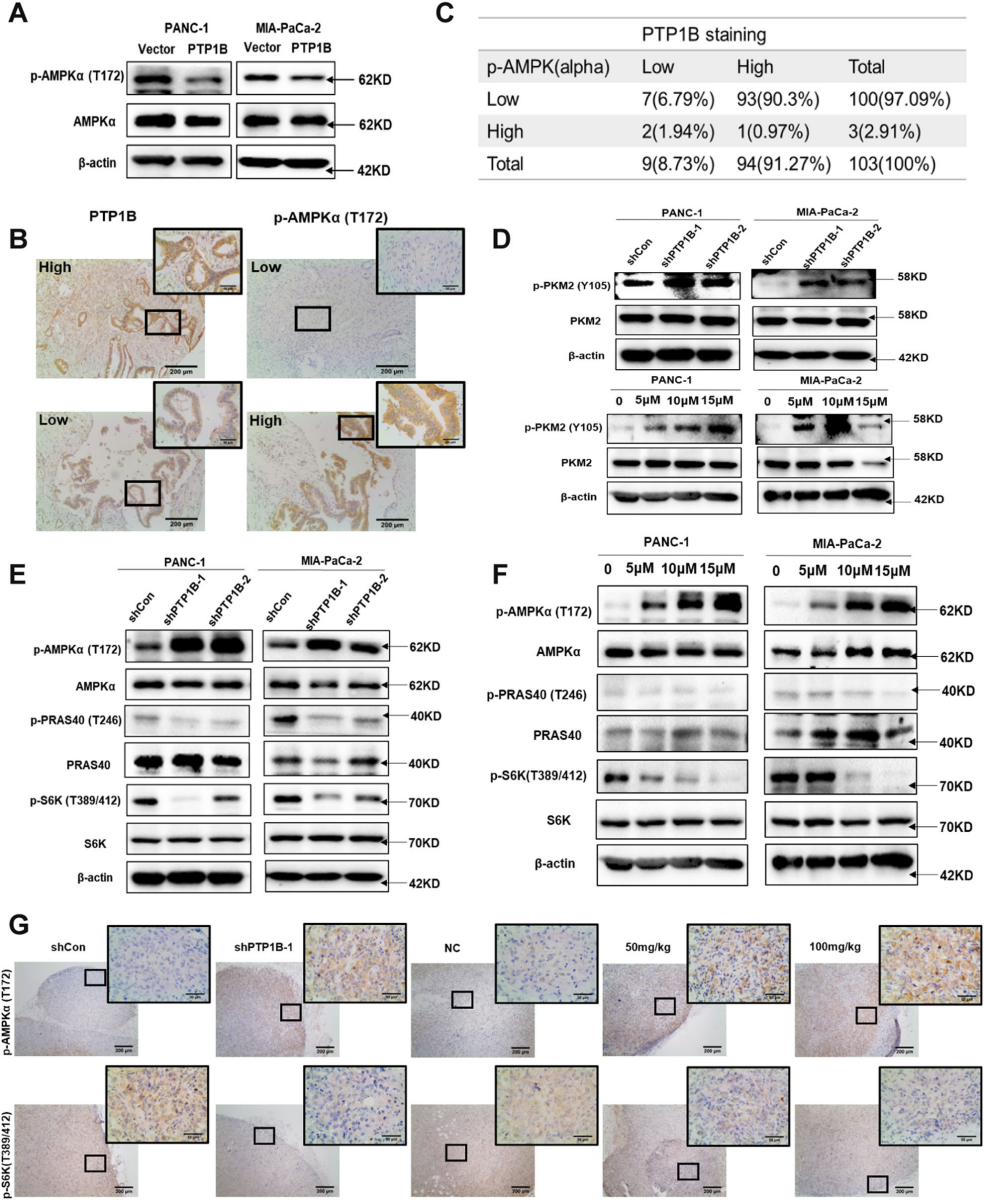
The relationship between PTP1B and AMPK. **a** PTP1B overexpression resulted in decreased p-AMPK (alpha). **b**, **c** The negative correlation between PTP1B and p-AMPKα was showed in pancreatic cancer patient tissue samples (*p* < 0.001, *p* value was obtained by a Pearson χ2 test; scale bar, 200 μm and 50 μm). **d** PTP1B inhibition either by shRNAs or by LXQ46 increased the phosphorylation of PKM2. **e**, **f** The inactivated PKM2 resulted in increased phosphorylation of AMPKα and decreased the phosphorylation of PRAS40, causing the inhibition of mTOC1 activity. **g** PTP1B inhibition caused AMPK activation and decreased p-p70S6K in vivo (scale bar, 200 and 50 μm).

**Fig. 7:**
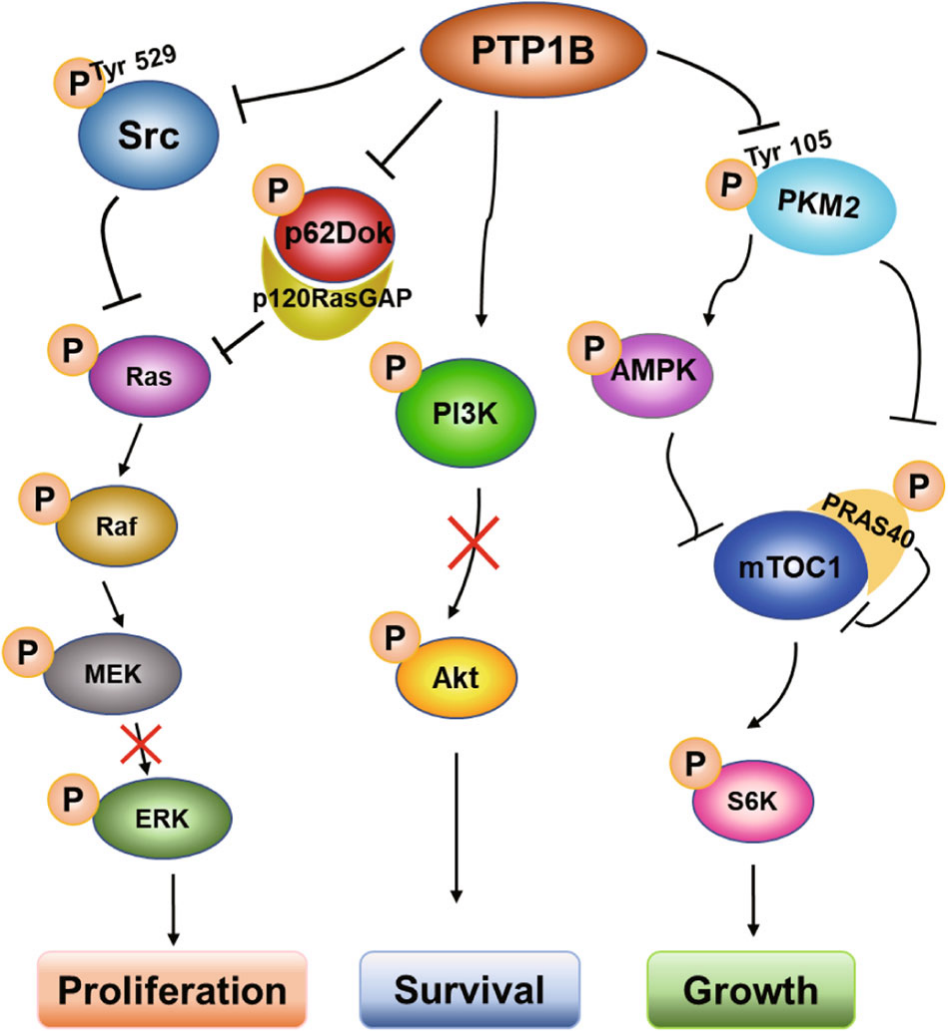
Proposed mechanism of pancreatic cancer cell proliferative arrest caused by PTP1B inhibition.

To elucidate the molecular mechanism of PTP1B in regulating the proliferation and growth of PDAC cells, we first focused on established PTP1B targeting molecular pathways that may contribute to tumor development, such as the Src/ERK and PI3K/Akt pathways^9^. Interestingly, the results showed that neither of these two signaling pathways was significantly inhibited in PTP1B-knockdown PDAC cells (Supplementary Fig. 4), indicating that PDAC cell growth suppression by PTP1B inhibition was associated with a different molecular pathway. Given that AMPK activation plays a critical role in cell cycle control and proliferation inhibition, along with our findings that p-AMPK was downregulated in pancreatic cancer (Supplementary Fig. 5), we next investigated the relationship between PTP1B and AMPK. The phosphorylation of AMPK was assessed in PTP1B overexpressing PDAC cell lines. As shown in Fig. 6a, the phosphorylation of AMPK was decreased when PTP1B was overexpressed, suggesting that PTP1B negatively regulates AMPK activation. Additionally, the negative association between PTP1B and p-AMPK was verified in pancreatic cancer tissues (Fig. 6b, c). Then, we demonstrated that PKM2, a substrate of PTP1B, mediates the interaction between PTP1B and AMPK. As shown in Fig. 6d, the activity of PKM2 was suppressed by its Tyr-105 phosphorylation in PTP1B deficiency PDAC cells. Consistently, the PTP1B inhibitor significantly increased PKM2 Tyr-105 phosphorylation and decreased PKM2 activity in a dose-dependent manner (Fig. 6d). Subsequently, the inhibition of PKM2 via either PTP1B knockdown or LXQ46 treatment activated AMPK in pancreatic cancer cells (Fig. 6e, f). In addition, the phosphorylation level of PRAS40 also decreased accompanied by decreased PKM2 activity (Fig. 6e, f). We then detected the level of p-p70S6K, a major downstream target of mTOC1, because the dephosphorylation of PRAS40 and AMPK activation could both indirectly inhibit mTOC1 activity to negatively regulate protein synthesis^17–19^. The results revealed that the phosphorylation of p70S6K was strongly repressed in response to the increased p-AMPK and decreased p-PRAS40 caused by PTP1B inhibition (Fig. 6e, f). The same results were obtained in xenograft tumor tissues (Fig. 6g). Taken together, these data demonstrated that PTP1B inhibition resulted in hyperphosphorylation of Tyr105 of PKM2 and weakened the activity of PKM2, causing increased phosphorylation of AMPK and decreased phosphorylation of PRAS40. These changes were followed by mTOC1 suppression and decreased p70S6K phosphorylation. All of these results eventually culminated in protein synthesis suppression and proliferative arrest (Fig. 7).

## Discussion

PTP1B, a member of the PTP family, acts as a well-established regulator of numerous signaling cascades^6^. In addition to insulin- and leptin-mediated signals, an increasing number of reports have shown that PTP1B plays a dual role in oncogenesis depending on the cancer type^9^. By contrast, much less is known about the role of PTP1B in pancreatic cancer. In the present study, we showed for the first time that knockdown or pharmacological inhibition of PTP1B reduced pancreatic cancer progression both in vitro and in vivo. On the one hand, PTP1B was significantly overexpressed in pancreatic cancer specimens was associated with distant metastasis and tumor staging, and indicated poor survival (Fig. 1 and Table 1), which demonstrated that PTP1B may play a role in promoting PDAC development. On the other hand, PTP1B knockdown resulted in proliferative arrest and suppression of migration in tumor cells, which, together with tumor growth inhibition in xenograft mouse model (Figs. 2, 5), indicated that PTP1B plays an oncogenic role in pancreatic cancer. The results of PTP1B overexpression were also consistent with the pro-oncogenic activity of PTP1B (Fig. 3). Additionally, pancreatic cancer cell proliferation and tumor growth were highly decreased when PTP1B was inhibited by a specific inhibitor, which supported the notion of PTP1B as a promising therapeutic target for PDAC (Figs. 4 and 5). These results were concordant with its role in other cancers, such as breast cancer, colon cancer, and non-small cell lung cancer, among others. Therefore, given the important role of PTP1B in pancreatic cancer development, the mechanism underlying its regulation of pancreatic cancer cell proliferation needs to be elucidated. Historically, PTP1B has been thought to promote tumorigenesis, growth, survival, and metastasis by interacting with several oncogenic substrates, such as p62DOK, Src, PITX1, and IRβ, in several human cancers^20–24^. Among these substrates, Src, PITX1 and p62DOK all control cell proliferation by regulating ERK. IRβ activates the PI3K/Akt signaling pathways, thereby promoting proliferation. However, in our studies, we could not prove that the loss of PTP1B led to attenuation of ERK and Akt activation (Supplementary Fig. 4). Hence, this interesting result led us to suppose that there could be a regulator of proliferation that is indirectly related to PTP1B and involved in pancreatic cancer development.

Emerging proofs suggest that PTP1B regulates the key modulator involved in energy balance. Then, we discovered that AMPK, a key regulator of energy homeostasis, is negatively regulated by PTP1B (Fig. 6a). Although there is evidence suggesting that AMPK might help cancer cells survive under certain circumstances^25^, there is more support in the literature for the notion that AMPK acts as a tumor suppressor by leading to cell growth inhibition and cell cycle arrest^26–29^. In particular, AMPK activation has proven to be an effective strategy to inhibit pancreatic cancer cell growth^30–32^. As shown in Fig. 6b, c, immunohistochemistry analysis revealed that PTP1B was negatively correlated with the phosphorylation level of AMPK in human pancreatic cancer tissues, which was consistent with the phenomenon observed in PDAC cell lines (Fig. 6e, f) and in xenograft tumors (Fig. 6g). Moreover, in Figs. 2e and 4e, the knockdown and pharmacological inhibition of PTP1B both induced G0/G1 phase cell cycle arrest in PDAC cell lines, which was an obvious manifestation of AMPK activation. Previous studies have demonstrated that loss of PTP1B protects against metabolic diseases such as myocardial anomalies, atherosclerotic plaque formation and diabetes by activating AMPK^33–35^, further confirming the correlation and interaction between PTP1B and AMPK. Subsequently, we found that AMPK activation appears to exert its antitumor effects through p70S6K inhibition in vitro (Fig. 6e, f) and in vivo (Fig. 6g). This is because AMPK activation inhibits mTORC1 signaling by phosphorylating TSC2^36^ or directly disrupting the combination between mTORC1 and Raptor^37^; thus, p70S6K-mediated inhibition of mTORC1 signaling prevents both purine and pyrimidine synthesis, and, consequently controls cell proliferation^18,38^.

However, notably, activation of AMPK is dependent on the phosphorylation of AMPKα at Thr-172. Thus, AMPK is not a direct downstream substrate of PTP1B. Next, we discovered that PKM2 mediated PTP1B-AMPK interaction. PKM2 has been identified as a substrate of PTP1B because PTP1B deficiency and pharmacological inhibition lead to increased PKM2 total tyrosine, and Tyr105 phosphorylation inhibits PKM2 pyruvate kinase activity in vitro and in vivo^39^. In addition, studies have reported that the stable knockdown of PKM2 activates AMPK in response to the perturbed ATP homeostasis^40^. As shown in Fig. 6d, the activity of PKM2 was indeed suppressed when PTP1B was inhibited, which was followed by AMPK activation. Meanwhile, the phosphorylation of PRAS40 was significantly decreased when PKM2 was inactivated, which aggravated the inhibition of mTOC1 (Fig. 6e, f). This is because the phosphorylation of PRAS40 by PKM2 releases PRAS40 from mTOC1, which results in the activation of mTOC1 signaling for oncogenic growth^41^. Hence, we conclude that PTP1B controls pancreatic cancer progression by simultaneously regulating AMPK and PRAS40 through PKM2.

Taken together, in present study, our results are the first to demonstrate the critical role of PTP1B in PDAC. PTP1B acts as an oncogene, and its inhibition activates AMPK and decreases the PRAS40 phosphorylation level by suppressing PKM2, which results in the inhibition of mTOC1. Inhibition of mTOC1, and thus p70S6K, leads to pancreatic cancer cell proliferative arrest. These results strongly suggest the potential of PTP1B as a therapeutic target in PDAC and the study value of a PTP1B inhibitor in the treatment of pancreatic cancer.

## Supplementary information

Supplementary figures legends

Supplementary table 1

Declaration of contributions to article

Supplementary Figure 1

Supplementary Figure 2

Supplementary Figure 3

Supplementary Figure 4

Supplementary Figure 5

## References

[1] Walsh N (2018). Agnostic pathway/gene set analysis of genome-wide association data identifies associations for pancreatic cancer. J. Natl Cancer Inst..

[2] Chakravarthy D (2018). Palmatine suppresses glutamine-mediated interaction between pancreatic cancer and stellate cells through simultaneous inhibition of survivin and COL1A1. Cancer Lett..

[3] Kamisawa T, Wood LD, Itoi T, Takaori K (2016). Pancreatic cancer. Lancet.

[4] Siegel RL, Miller KD, Jemal A (2015). Cancer Statistics, 2015. Ca-Cancer J. Clin..

[5] Forsell PKAL, Boie Y, Montalibet J, Collins S, Kennedy BP (2000). Genomic characterization of the human and mouse protein tyrosine phosphatase-1B genes. Gene.

[6] Feldhammer M, Uetani N, Miranda-Saavedra D, Tremblay ML (2013). PTP1B: A simple enzyme for a complex world. Crit. Rev. Biochem. Mol. Biol..

[7] Krishnan N (2018). Harnessing insulin- and leptin-induced oxidation of PTP1B for therapeutic development. Nat. Commun..

[8] Penafuerte C (2017). Downregulation of PTP1B and TC-PTP phosphatases potentiate dendritic cell-based immunotherapy through IL-12/IFN signaling. Oncoimmunology.

[9] Bollu LR, Mazumdar A, Savage MI, Brown PH (2017). Molecular pathways: targeting protein tyrosine phosphatases in cancer. Clin. Cancer Res..

[10] Lessard L, Stuible M, Tremblay ML (2010). The two faces of PTP1B in cancer. Biochim. Biophys. Acta—Proteins Proteom..

[11] Arias-Romero LE (2009). Activation of src by protein tyrosine phosphatase 1B is required for ErbB2 transformation of human breast epithelial cells. Cancer Res..

[12] Krishnan N (2014). Targeting the disordered C terminus of PTP1B with an allosteric inhibitor. Nat. Chem. Biol..

[13] Fan GF, Lin G, Lucito R, Tonks NK (2013). Protein-tyrosine Phosphatase 1B antagonized signaling by insulin-like growth factor-1 receptor and kinase BRK/PTK6 in ovarian cancer cells. J. Biol. Chem..

[14] Dube N (2005). Genetic ablation of protein tyrosine phosphatase 1B accelerates lymphomagenesis of p53-null mice through the regulation of B-cell development. Cancer Res..

[15] Ji SR (2016). ALDOA functions as an oncogene in the highly metastatic pancreatic cancer. Cancer Lett..

[16] Li X (2019). Toward a treatment of diabesity: in vitro and in vivo evaluation of uncharged bromophenol derivatives as a new series of PTP1B inhibitors. Eur. J. Med Chem..

[17] Lu J (2013). Pim2 is required for maintaining multiple myeloma cell growth through modulating TSC2 phosphorylation. Blood.

[18] Kim LC, Cook RS, Chen J (2017). mTORC1 and mTORC2 in cancer and the tumor microenvironment. Oncogene.

[19] Lv D, Guo LY, Zhang T, Huang L (2017). PRAS40 signaling in tumor. Oncotarget.

[20] Tai WT (2016). Protein tyrosine phosphatase 1B dephosphorylates PITX1 and regulates p120RasGAP in hepatocellular carcinoma. Hepatology.

[21] Liu HB (2015). PTP1B promotes cell proliferation and metastasis through activating src and ERK1/2 in non-small cell lung cancer. Cancer Lett..

[22] Mertins P (2008). Investigation of protein-tyrosine phosphatase 1B function by quantitative proteomics. Mol. Cell. Proteom..

[23] Dube N, Cheng A, Tremblay ML (2004). The role of protein tyrosine phosphatase 1B in Ras signaling. Proc. Natl Acad. Sci. USA.

[24] Bakke J, Haj FG (2015). Protein-tyrosine phosphatase 1B substrates and metabolic regulation. Semin. Cell Dev. Biol..

[25] Hardie DG (2015). Molecular pathways: is AMPK a friend or a foe in cancer?. Clin. Cancer Res..

[26] Penfold L (2018). CAMKK2 promotes prostate cancer independently of AMPK via increased lipogenesis. Cancer Res..

[27] Wu F (2018). miR-1273g silences MAGEA3/6 to inhibit human colorectal cancer cell growth via activation of AMPK signaling. Cancer Lett..

[28] Carling D (2017). AMPK signalling in health and disease. Curr. Opin. Cell Biol..

[29] Faubert B (2013). AMPK is a negative regulator of the warburg effect and suppresses tumor growth in vivo. Cell Metab..

[30] Xu C (2019). Targeting surface nucleolin induces autophagy-dependent cell death in pancreatic cancer via AMPK activation. Oncogene.

[31] Chen X (2018). Activation of Nrf2 by sulforaphane inhibits high glucose-induced progression of pancreatic cancer via AMPK dependent signaling. Cell. Physiol. Biochem..

[32] Duan WX (2017). Desmoplasia suppression by metformin-mediated AMPK activation inhibits pancreatic cancer progression. Cancer Lett..

[33] Kandadi MR (2015). Deletion of protein tyrosine phosphatase 1B rescues against myocardial anomalies in high fat diet-induced obesity: Role of AMPK-dependent autophagy. Biochim. Biophys. Acta—Mol. Basis Dis..

[34] Thompson D (2017). Myeloid protein tyrosine phosphatase 1B (PTP1B) deficiency protects against atherosclerotic plaque formation in the ApoE (-/-) mouse model of atherosclerosis with alterations in IL10/AMPK alpha pathway. Mol. Metab..

[35] Yang JL, Ha TKQ, Lee BW, Kim J, Oh WK (2017). PTP1B inhibitors from the seeds of Iris sanguinea and their insulin mimetic activities via AMPK and ACC phosphorylation. Bioorg. Medicinal Chem. Lett..

[36] Inoki K, Zhu TQ, Guan KL (2003). TSC2 mediates cellular energy response to control cell growth and survival. Cell.

[37] Gwinn DM (2008). AMPK phosphorylation of raptor mediates a metabolic checkpoint. Mol. Cell.

[38] Morran DC (2014). Targeting mTOR dependency in pancreatic cancer. Gut.

[39] Bettaieb A (2013). Protein tyrosine phosphatase 1B regulates pyruvate kinase M2 tyrosine phosphorylation. J. Biol. Chem..

[40] Prakasam G (2017). Pyruvate kinase M knockdown-induced signaling via AMP-activated protein kinase promotes mitochondrial biogenesis, autophagy, and cancer cell survival. J. Biol. Chem..

[41] He CL (2016). Pyruvate kinase M2 activates mTORC1 by phosphorylating AKT1S1. Sci. Rep..

