# Supplementary material for: Inhibition of PTP1B blocks pancreatic cancer progression by targeting the PKM2/AMPK/mTOC1 pathway
Source: Cell Death Dis. 2019 Nov 19;10(12):874. doi: 10.1038/s41419-019-2073-4 (PMC6864061; doi:10.1038/s41419-019-2073-4)
Supplement: Supplementary file 1 — Supplementary figures legends [file 41419_2019_2073_MOESM1_ESM.docx]

**Supplementary Figure 1.** The effect of LV3-shRNAs on pancreatic cancer cell growth. (A and B) The cell number of control and PTP1B knockdown groups in PANC-1 and MIA-PaCa-2 cells after LV3-shRNAs transfection (scale bar, 100 μm). Cells in negative control groups were set as 100% (reference). All the quantitative data are represented as mean ± SEM of three independent experiments and **p*＜0.05, ***p*＜0.01 versus control group.

**Supplementary Figure 2.** Apoptosis was not the main reason for the cell proliferation sluggish caused by PTP1B inhibition. (A) PTP1B knockdown did not affect apoptosis. The apoptosis-related proteins in PTP1B-knockdown PANC-1 cells were examined by Western blotting. (B) PTP1B inhibition by LXQ46 did not induce apoptosis. PANC-1 cells were treated with indicated concentrations of LXQ46 (0, 5, 10, 15μM) for 48 h, then Bcl-xL, PARP, Bcl-2 and Bax were detected by Western blotting.

**Supplementary Figure 3.** The effect of LXQ46 on pancreatic cancer cells proliferation. (A) Western blot determined that LXQ46 did not inhibit PTP1B expression. (B-E) MTT assays showed LXQ46 significantly reduced the viability of PDAC cell lines. LXQ46 inhibited AsPC-1, BxPC-3, SW-1990 and Panc-28 cells proliferation with IC_50_ of 7.16 μM, 3.15 μM, 6.9 μM and 4.89 μM. All experiments were performed in triplicate. **p* < 0.05, ***p* < 0.01 versus control group.

**Supplementary Figure 4.** PTP1B knockdown prevented pancreatic cancer cell growth not through Src/ERK or PI3K/Akt pathways. The antibodies against p-Src (Y529), total Src, p-ERK (T202/Y204), total ERK, p-PI3K (Y607), total PI3K, p-Akt (S473) and total Akt were used to determine the effect of PTP1B on the activities of Src/ERK and PI3K/Akt pathways.

**Supplementary Figure 5.** The level of p-AMPK was downregulated in tumor tissues compared with normal pancreas. (A) The immunohistochemistry (IHC) assay of p-AMPKα in 13 samples of normal pancreas and 103 samples of PDAC tissues (scale bar, 200 μm and 50 μm). (B) Staining index of p-AMPKα in tumor and non-neoplastic tumor tissue. **p* < 0.05 and ***p* < 0.01 compared with control.
