# Supplementary material for: Inhibition of PTP1B blocks pancreatic cancer progression by targeting the PKM2/AMPK/mTOC1 pathway
Source: Cell Death Dis. 2019 Nov 19;10(12):874. doi: 10.1038/s41419-019-2073-4 (PMC6864061; doi:10.1038/s41419-019-2073-4)
Supplement: Supplementary file 2 — Supplementary table 1 [file 41419_2019_2073_MOESM2_ESM.docx]

**Supplementary Table 1.** Clinical characteristics of PDAC patients.

| **Characteristic** | **n** |
| --- | --- |
| Number of patients | 118 |
| Gender |  |
| Male | 71 (60.2%) |
| Female | 47 (39.8%) |
| Age |  |
| ＜60 | 49 (41.5%) |
| ≥60 | 69 (58.5%) |
| Tumor (Topography) |  |
| T1,＜2cm | 23 (19.5%) |
| T2, ≥2cm,≤4 | 67 (56.8%) |
| T3,＞4 | 21 (17.8%) |
| T4, invasion | 7 (5.9%) |
| Lymph node metastasis (N) |  |
| NO | 80 (67.8%) |
| YES | 38 (32.2%) |
| Distant metastasis (M) |  |
| NO | 107 (90.7%) |
| YES | 11 (9.3%) |
| Differentiation |  |
| Low | 28 (23.7%) |
| Medium | 86 (72.9%) |
| High | 4 (3.4%) |
| Tumor staging |  |
| I | 42 (35.6%) |
| II | 59 (50%) |
| III | 7 (5.9%) |
| IV | 10 (8.5%) |
