# Supplementary material for: Inhibition of PTP1B blocks pancreatic cancer progression by targeting the PKM2/AMPK/mTOC1 pathway
Source: Cell Death Dis. 2019 Nov 19;10(12):874. doi: 10.1038/s41419-019-2073-4 (PMC6864061; doi:10.1038/s41419-019-2073-4)
Supplement: Supplementary file 3 — Declaration of contributions to article [file 41419_2019_2073_MOESM3_ESM.pdf]

DECLARATION OF CONTRIBUTIONS TO ARTICLE

ADMC

Manuscript Number:	Journal Name:	
CDDIS-19-1203	*Cell Death & Disease*	(the 'Journal')

Proposed Title of the Contribution:

Author contributions to "Inhibition of PTP1B blocks pancreatic cancer progression by targeting the PKM2/AMPK/mTOC1 pathway"	(the 'Contribution)

Author(s):

Qi Xu, Ning Wu, Xiangqian Li, Chuanlong Guo, Chao Li, Bo Jiang, Huaizhi Wang, Dayong Shi	(the 'Authors')

For all *CDDis* articles, each person named as an author in the published version must be able to show he or she has contributed substantially to the article.

Authorship credit should be based on 1) substantial contributions to conception and design, acquisition of data, or analysis and interpretation of data; 2) drafting the article or revising it critically for important intellectual content; and 3) final approval of the version to be published. Authors should meet conditions 1, 2 and 3.

Any person who cannot be shown to have made a substantial contribution to the article cannot be listed as an author in the final version. The name of any person who is deemed to have made a minor contribution can, however, appear in the Acknowledgments section of the article.

Please complete the table below to indicate the contributions of all named authors to the manuscript.

Author Full Name:	Specification of Contribution to the Manuscript:
Qi Xu	Acquisition of data; drafting the article; final approval of the version to be published
Ning Wu	Analysis and interpetation of data; drafting the article; final approval of the version to be published
Xiangqian Li	Acquisition of data; revising draft critically for important intellectual content; final approval of the version to be published
Chuanlong Guo	Acquisition of data; analysis and interpretation of data; final approval of the version to be published
Chao Li	Analysis and interpretation of data; revising draft critically for important intellectual content; final approval of the version to be published
Bo Jiang	Analysis and interpretation of data; analysis and interpretation of data; final approval of the version to be published
Huaizhi Wang	Contribution to conception and design; revising draft critically for important intellectual content; final approval of the version to be published
Dayong Shi	Contribution to conception and design, acquisition of data; final approval of the version to be published

Please complete the table below to indicate the contributions of all named authors to the figures.

Figure 1:
Qi Xu; Huaizhi Wang; Ning Wu; Chuanlong Guo

Figure 2:
Qi Xu; Chuanlong Guo; Ning Wu; Chao Li

Figure 3:
Qi Xu, Ning Wu, Chao Li, Bo Jiang

Figure 4:
Xiangqian Li; Dayong Shi; Bo jiang; Chao Li

Figure 5:
Qi Xu, Ning Wu, Chuanlong Guo; Xiangqian Li

Figure 6:
Qi Xu; Ning Wu, Dayong Shi, Huaizhi Wang

Signed for and on behalf of the Author(s):
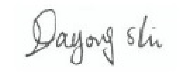

Print Name: Dayong Shi

Date: April 2, 2019
