# Supplementary figures and images for: Inhibition of PTP1B blocks pancreatic cancer progression by targeting the PKM2/AMPK/mTOC1 pathway
Source: Cell Death Dis. 2019 Nov 19;10(12):874. doi: 10.1038/s41419-019-2073-4 (PMC6864061; doi:10.1038/s41419-019-2073-4)

# Supplementary Fig. 1

**A**

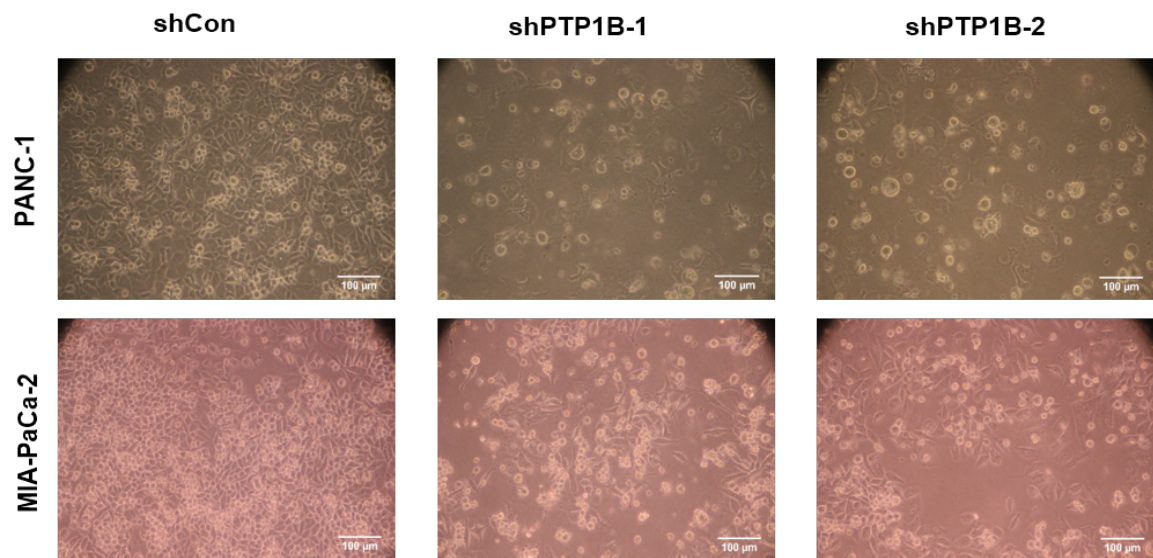

**B**

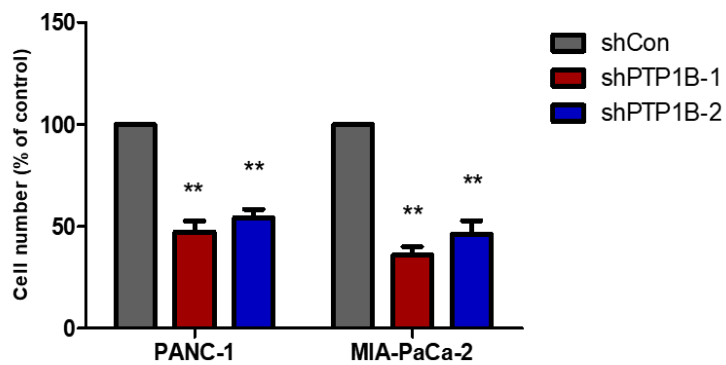

Supplement: Supplementary file 4 — Supplementary Figure 1 [file 41419_2019_2073_MOESM4_ESM.pdf]

## Supplementary Fig.2

**A**

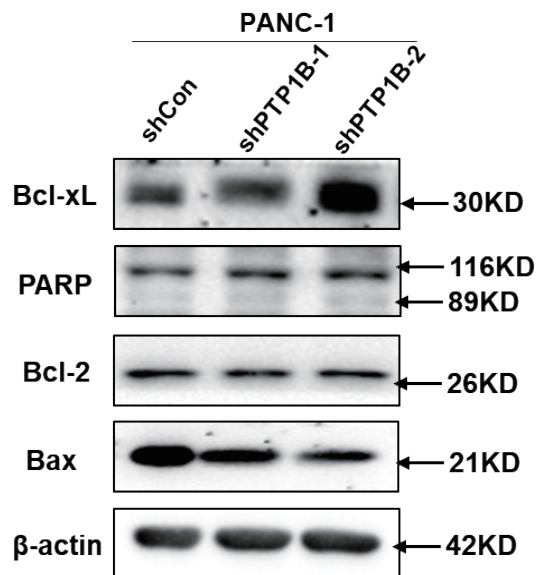

**B**

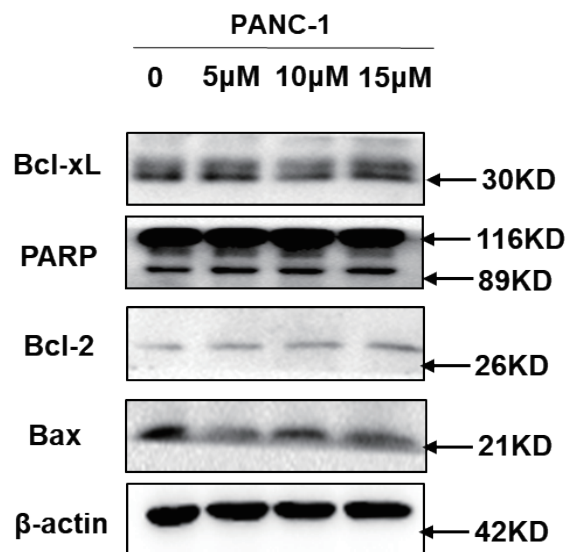

Supplement: Supplementary file 5 — Supplementary Figure 2 [file 41419_2019_2073_MOESM5_ESM.pdf]

# Supplementary Fig. 3

**A**

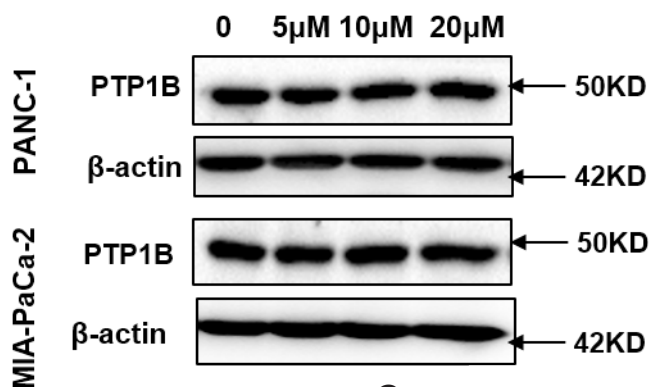

**B**

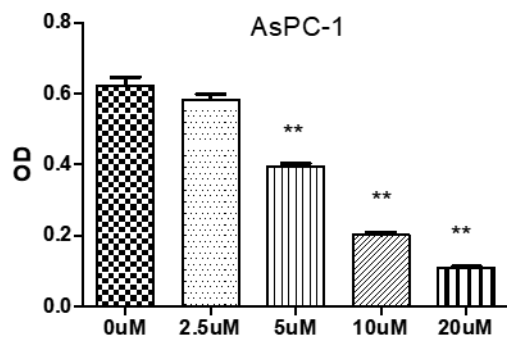

**C**

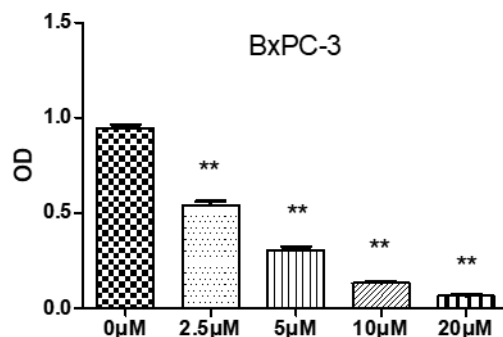

**D**

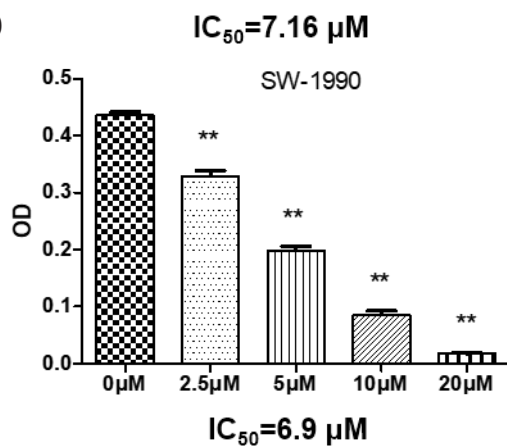

**E**

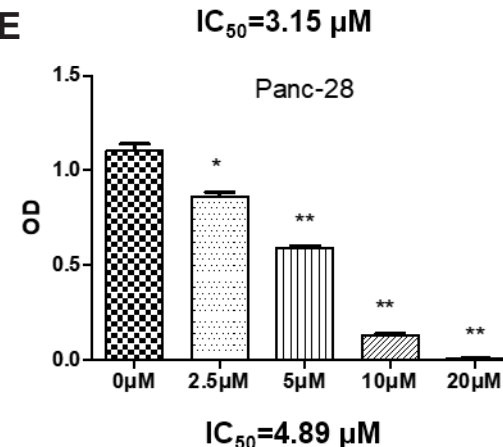

Supplement: Supplementary file 6 — Supplementary Figure 3 [file 41419_2019_2073_MOESM6_ESM.pdf]

## Supplementary Fig.4

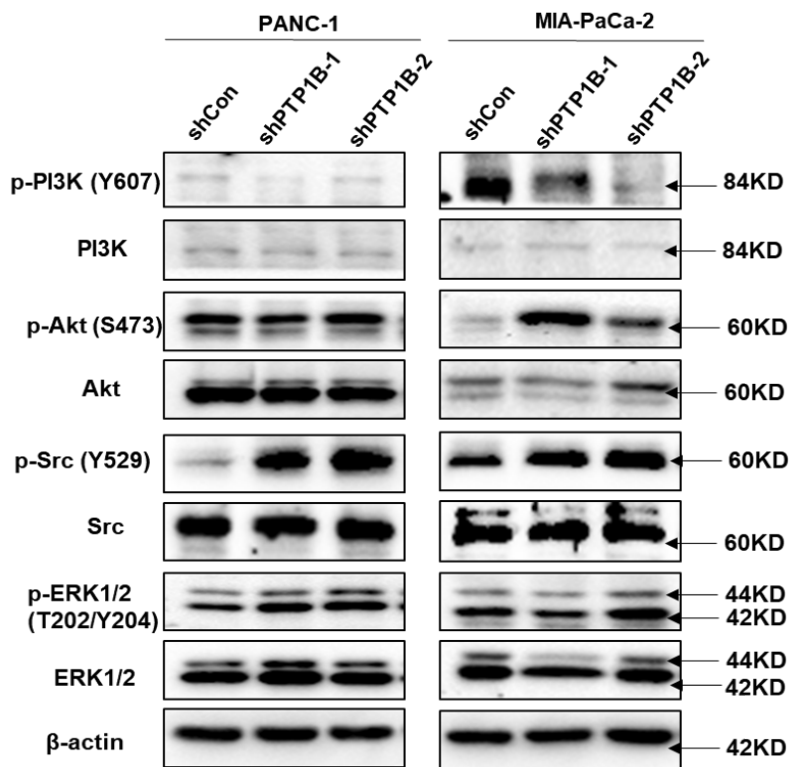

Supplement: Supplementary file 7 — Supplementary Figure 4 [file 41419_2019_2073_MOESM7_ESM.pdf]

## Supplementary Fig.5

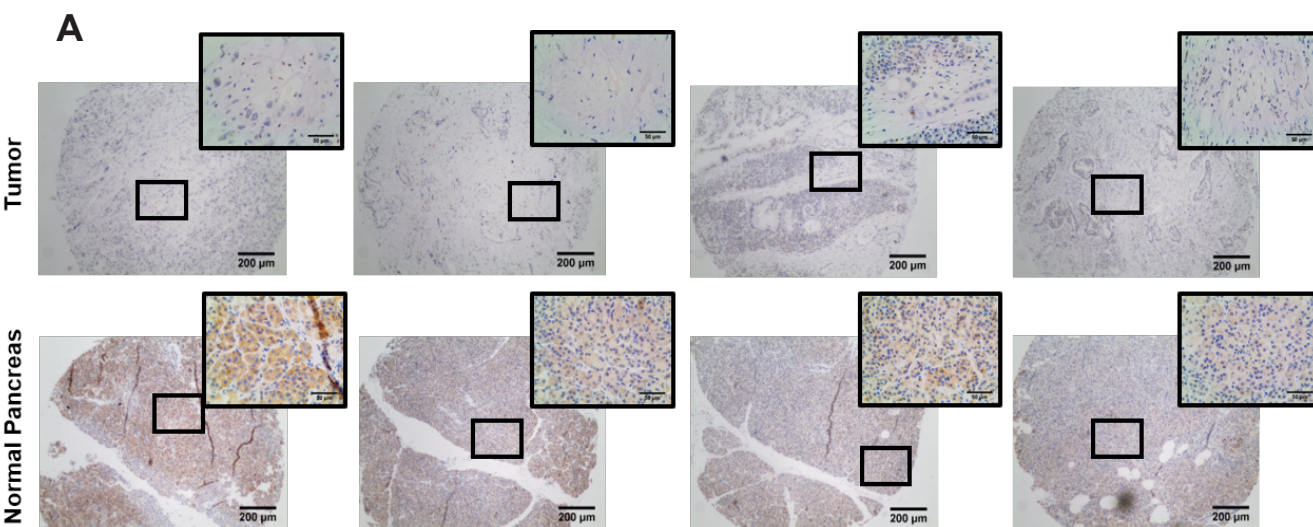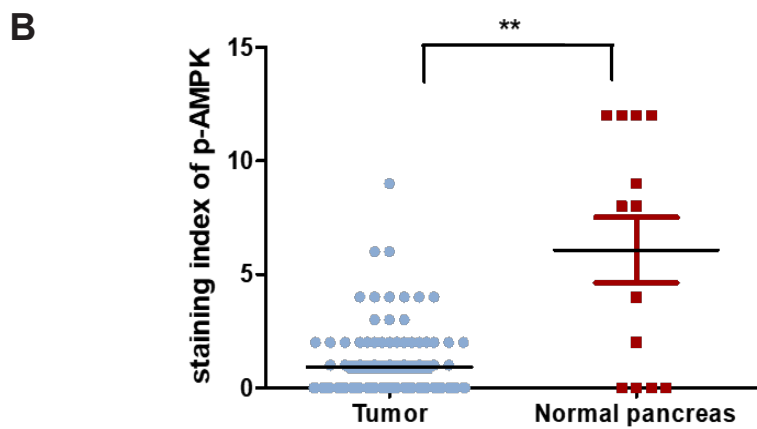

Supplement: Supplementary file 8 — Supplementary Figure 5 [file 41419_2019_2073_MOESM8_ESM.pdf]
